# Supplementary material for: Verification of the Vitek Reveal System for Direct Antimicrobial Susceptibility Testing in Gram-Negative Positive Blood Cultures
Source: Antibiotics (Basel). 2024 Nov 7;13(11):1058. doi: 10.3390/antibiotics13111058 (PMC11590937; doi:10.3390/antibiotics13111058)
Supplement: Supplementary file 1 [file antibiotics-13-01058-s001.zip › antibiotics-3285478-supplementary.pdf]

Table S1. Distribution of β-lactam resistance genes across bacterial isolates used for spiking blood culture bottles

Bacterial isolates		Detected gene(s) in bacterial isolates
Sequential number	Species	
1	*Acinetobacter baumannii*	bla_{NDM-1}
2	*A. baumannii*	bla_{NDM-1}
3	*A. baumannii*	bla_{NDM-1}
4	*A. baumannii*	bla_{NDM-1}
5	*A. baumannii*	bla_{NDM-1}
6	*A. baumannii*	bla_{NDM-1}
7	*Escherichia coli*	$bla_{CTX-M-15}$
8	*E. coli*	bla_{CMY-2}
9	*E. coli*	bla_{CMY-2}
10	*E. coli*	bla_{CMY-2}
11	*E. coli*	bla_{DHA-1}
12	*E. coli*	bla_{DHA-1}
13	*E. coli*	bla_{DHA-1}
14	*E. coli*	bla_{SHV-12}
15	*E. coli*	$bla_{CTX-M-15}$
16	*E. coli*	$bla_{CTX-M-15}$
17	*E. coli*	$bla_{CTX-M-15}$
18	*E. coli*	$bla_{CTX-M-15}$
19	*E. coli*	bla_{CMY-2}
20	*Klebsiella aerogenes*	$bla_{CTX-M-3}$
21	*K. aerogenes*	$bla_{OXA-181}$
22	*Klebsiella oxytoca*	bla_{DHA-1}
23	*Klebsiella pneumoniae*	bla_{KPC-3}
24	*K. pneumoniae*	$bla_{CTX-M-15}$, bla_{NDM-1}
25	*K. pneumoniae*	$bla_{CTX-M-15}$, bla_{NDM-1}
26	*K. pneumoniae*	bla_{KPC-3}, bla_{VIM-1}
27	*K. pneumoniae*	bla_{KPC-3}
28	*K. pneumoniae*	$bla_{CTX-M-15}$, bla_{KPC-3}
29	*K. pneumoniae*	$bla_{CTX-M-15}$, bla_{KPC-31}
30	*K. pneumoniae*	$bla_{CTX-M-15}$, bla_{KPC-31}
31	*K. pneumoniae*	bla_{KPC-31}
32	*K. pneumoniae*	bla_{KPC-31}
33	*K. pneumoniae*	bla_{CMY-2}, bla_{KPC-3}
34	*K. pneumoniae*	bla_{DHA-1}
35	*K. pneumoniae*	bla_{SHV-12}
36	*K. pneumoniae*	bla_{SHV-12}
37	*K. pneumoniae*	$bla_{CTX-M-15}$

38	*K. pneumoniae*	bla_{KPC-3}; bla_{VIM-1}
39	*P. aeruginosa*	None
40	*P. aeruginosa*	None
41	*P. aeruginosa*	None
42	*P. aeruginosa*	bla_{VIM-2}
43	*P. aeruginosa*	bla_{VIM-2}
44	*P. aeruginosa*	bla_{VIM-2}
45	*P. aeruginosa*	bla_{IMP-13}
46	*P. aeruginosa*	bla_{IMP-13}
47	*P. aeruginosa*	bla_{IMP-13}
48	*P. aeruginosa*	bla_{GES-2}
49	*P. aeruginosa*	None
50	*Proteus mirabilis*	bla_{CMY-16}

Note: The bacterial organisms listed here belong to a hospital collection of clinical isolates that were characterized using a well-established PCR-sequencing approach. "None" denotes the absence of any β-lactam resistance genes investigated. Excluding *A. baumannii* complex (*n* = 6) or *P. aeruginosa* (*n* = 11) organisms, the remaining organisms were all *Enterobacterales*, such as *E. coli* (*n* = 13), *K. aerogenes* (*n* = 2), *K. oxytoca* (*n* = 1), *K. pneumoniae* (*n* = 16) and *P. mirabilis* (*n* = 1).

Table S2. Antibiotics and their concentrations used in the Vitek Reveal and reference AST assays

	Vitek Reveal	Reference	
Antibiotics	Range of concentrations (µg/mL)	Range of concentrations (µg/mL)	Suppliers of antibiotic powders used to prepare working dilutions
Amikacin	4–16	2–32	Sigma Aldrich
Amoxicillin/clavulanic acid	4–16	2–64	USP reference standard (amoxicillin) and Sigma Aldrich (potassium clavulanate)
Ampicillin	4–8	2–16	Sigma Aldrich
Aztreonam	1–16	0.5–32	Sigma Aldrich
Cefepime	0.125–64	0.06–128	USP reference standard
Cefotaxime	0.125–4	0.06–8	Sigma Aldrich
Ceftazidime	0.125–64	0.06–128	Sigma Aldrich
Ceftazidime/avibactam	0.25–16	0.12–32	Sigma Aldrich
Ceftolozane/tazobactam	1–4	0.5–8	Sigma Aldrich
Ciprofloxacin	0.06–1	0.03–2	Sigma Aldrich
Ertapenem	0.125–1	0.06–2	Sigma Aldrich
Gentamicin	2–4	1–8	Sigma Aldrich
Imipenem	1–8	0.5–16	Sigma Aldrich
Levofloxacin	0.25–1	0.12–2	Toku-E
Meropenem	0.125–8	0.06–16	Sigma Aldrich
Meropenem/vaborbactam	2–8	1–16	Sigma Aldrich (meropenem) and MedChem express (vaborbactam)
Piperacillin	8–16	4–32	Sigma Aldrich
Piperacillin/tazobactam	4–16	2–32	Sigma Aldrich
Tigecycline	0.5–1	0.25–2	Sigma Aldrich
Tobramycin	2–4	1–8	Sigma Aldrich
Trimethoprim/sulfamethoxazole	2–4	1–8	Sigma Aldrich

Table S3. Combinations of antimicrobial agents and Gram-negative bacterial organisms tested with the Vitek Reveal AST assay

	Organisms stratified by species							
Antimicrobial agents	*Acinetobacter baumannii*	*Enterobacter cloacae*	*Escherichia coli*	*Klebsiella aerogenes*	*Klebsiella oxytoca*	*Klebsiella pneumoniae*	*Proteus mirabilis*	*Pseudomonas aeruginosa*
Amikacin	●	●	●	●	●	●	●	●
Amoxicillin/clavulanic acid			●		●	●	●	
Ampicillin			●				●	
Aztreonam		●	●	●	●	●		●
Cefepime		●	●		●	●	●	●
Cefotaxime		●	●	●	●	●	●	
Ceftazidime		●	●	●	●	●	●	●
Ceftazidime/avibactam		●	●	●	●	●	●	●
Ceftolozane/tazobactam		●	●	●	●	●		●
Ciprofloxacin	●	●	●	●	●	●	●	●
Ertapenem		●	●	●	●	●	●	
Gentamicin	●	●	●	●	●	●	●	
Imipenem	●	●	●	●	●	●		●
Levofloxacin	●	●	●	●	●	●	●	●
Meropenem	●	●	●	●	●	●	●	●
Meropenem/vaborbactam		●	●	●	●	●	●	●
Piperacillin		●	●	●	●	●	●	●
Piperacillin/tazobactam		●	●	●	●	●	●	●
Tigecycline			●					
Tobramycin	●	●	●	●	●	●	●	●
Trimethoprim/sulfamethoxazole	●	●	●	●	●	●	●	
Total by species	8	18	21	17	19	19	17	14

Note: Of all antibiotic/organism pairs for which a minimum inhibitory concentration (MIC) result can be determined, the Vitek Reveal (bioMérieux, Marcy l'Étoile, France) system is designed to provide MIC results only for those pairs where EUCAST breakpoints are available and for which intrinsic resistance to the included antimicrobials is unknown (indicated here by a dot). For example, no MIC result is reported for the piperacillin/tazobactam and *A. baumannii* combination, while MICs of meropenem against all seven bacterial species (including *A. baumannii*) included in this study are reported.

(a)

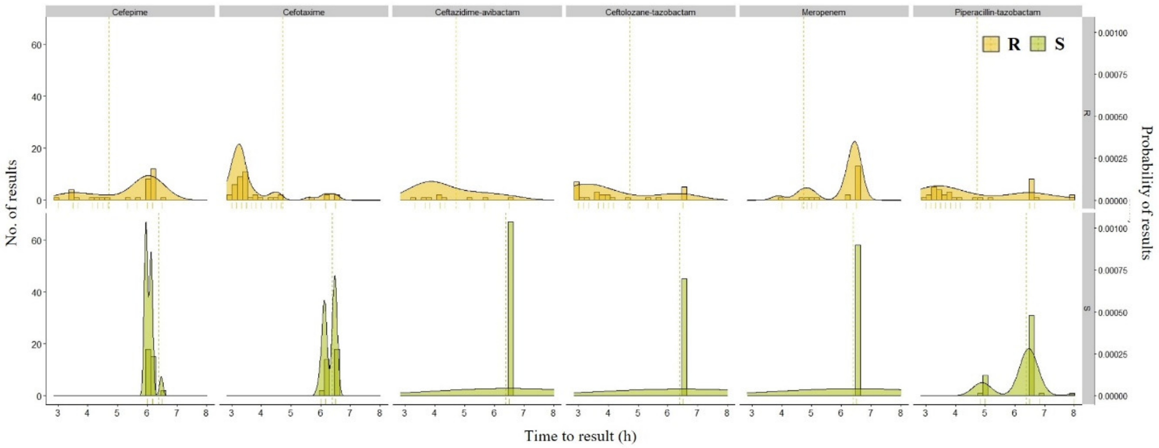

	Cefepime		Cefotaxime		Ceftazidime/avibactam		Ceftolozane/tazobactam		Meropenem		Piperacillin/tazobactam	
	R	S	R	S	R	S	R	S	R	S	R	S
No. of results	32	37	40	34	9	67	27	44	13	59	34	42
Time to results (h)												
Mean	05:23	06:06	03:51	06:20	04:32	06:30	04:15	06:30	06:01	06:30	04:44	06:13
SD	01:05	00:07	00:58	00:11	01:02	00:00	01:20	00:00	00:47	00:00	01:34	00:41
p-value	0.0097		<0.0001		0.0006		<0.0001		0.1705		<0.0001	

(b)

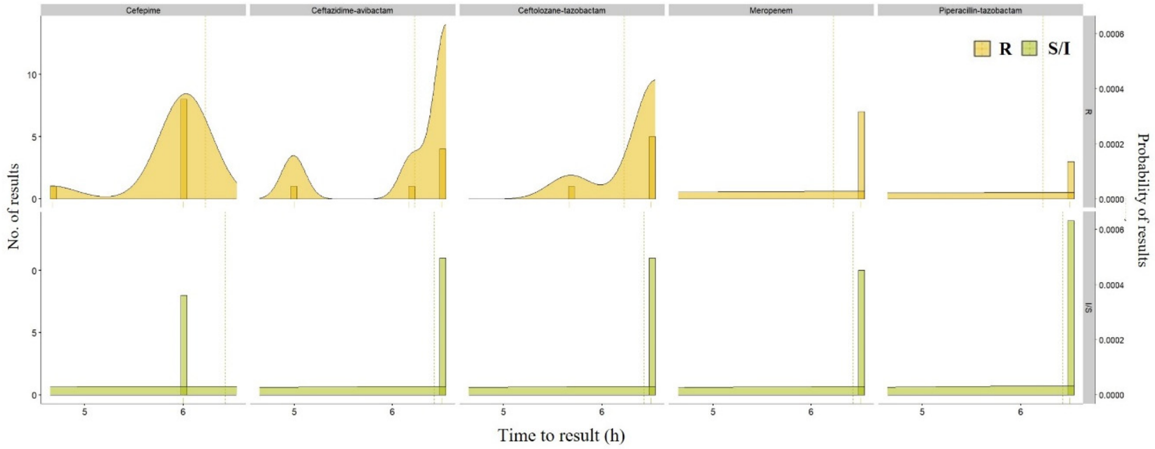

	Cefepime		Ceftazidime/avibactam		Ceftolozane/tazobactam		Meropenem		Piperacillin/tazobactam	
	R	S/I	R	S/I	R	S/I	R	S/I	R	S/I
No. of results	5	12	6	11	6	11	8	9	8	9
Time to results										
Mean	05:51	06:00	06:12	06:30	06:22	06:30	06:30	06:30	06:30	06:30
SD	00:27	00:00	00:36	00:00	00:20	00:00	00:00	00:00	00:00	00:00
p-value	0.3466		0.2676		0.3632		NA		NA	

Figure S1. Times to MIC results by Vitek Reveal AST assay for *Enterobacterales* (a) and *Pseudomonas aeruginosa* (b) organisms from positive blood culture samples and selected antibiotics tested. Times (range: 3 to 8 hours) were stratified according to the S/I/R groups of organisms and presented using histograms with Kernel density plots. Differences between groups (in terms of means ± standard deviation) were assessed for statistical significance. The probability of MIC results falling in the indicated range was calculated by fixing the total area under the curve of each density plot equal to 1.

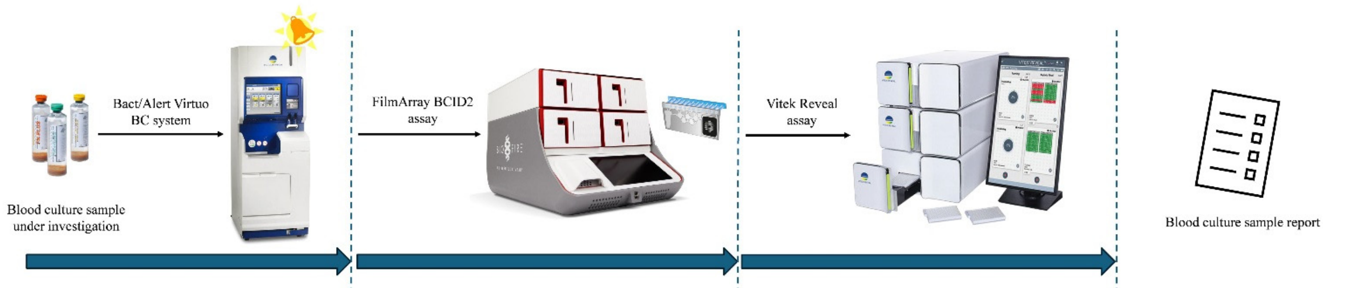

Time to positivity (T_1)	
Mean (h)	08:52:16
SD (h)	02:45:05
Min value (h)	04:48:00
Max value (h)	14:54:00

Time to identification (T_2)		
	Hands-on time	Time to assay result
Mean (h)	00:04:44	01:05:00
SD (h)	00:00:53	00:00:00
Min value (h)	00:04:32	01:05:00
Max value (h)	00:05:40	01:05:00

Time to AST result (T_3)		
	Hands-on time	Time to assay result
Mean (h)	00:03:47	06:31:00
SD (h)	00:00:28	00:25:43
Min value (h)	00:03:26	06:30:00
Max value (h)	00:03:47	06:30:00

Time to report ($T_1 + T_2 + T_3$)	
Mean (h)	16:36:46
SD (h)	02:50:36
Min value (h)	12:32:27
Max value (h)	22:36:58

Figure S2. Times from sampling of clinical blood cultures (*n* = 50) for microbiological investigation to reporting of results. Three intervals (expressed as means ± standard deviation) are shown: the time until a blood culture was detected as positive for a Gram-negative organism (T_1); the time until a FilmArray BCID2 assay-based identification result was generated for the organism (T_2); and the time until a Vitek Reveal assay-based AST result was generated for the organism (T_3). The T_2 and T_3 intervals were further subdivided into the hands-on time and the time to assay result. The total time to report was obtained by summing T_1, T_2 and T_3.
